# New Biocompatible Mesoporous Silica/Polysaccharide Hybrid Materials as Possible Drug Delivery Systems

**DOI:** 10.3390/ma12010015

**Published:** 2018-12-20

**Authors:** Andreea Madalina Pandele, Corina Andronescu, Adi Ghebaur, Sorina Alexandra Garea, Horia Iovu

**Affiliations:** 1 Advanced Polymer Materials Group, Faculty of Applied Chemistry and Material Science, University Polytehnica of Bucharest, str. Gheorghe Polizu 1-7, 0011601 Bucharest, Romania; pandele.m.a@gmail.com (A.M.P.); Corinaandronescu@yahoo.com (C.A.); ghebauradi@yahoo.com (A.G.); garea_alexandra@yahoo.co.uk (S.A.G.); 2 Analytical Chemistry–Center for Electrochemical Science, Ruhr–Universitat Bochum, Universitätstraße 150, D-44780 Bochum, Germany; 3 Academy of Romanian Scientist, 0011601 Bucharest, Romania

**Keywords:** MSN, biopolymer, drug delivery system, in vitro kinetic studies

## Abstract

A high number of studies support the use of mesoporous silica nanoparticles (MSN) as carriers for drug delivery systems due to its high biocompatibility both in vitro and in vivo, its large surface area, controlled pore size and, more than this, its good excretion capacity from the body. In this work we attempt to establish the optimal encapsulation parameters of benzalkonium chloride (BZC) into MSN and further study its drug release. The influence of different parameters towards the drug loading in MSN such as pH, contact time and temperature were considered. The adsorption mechanism of the drug has been determined by using the equilibrium data. The modification process was proved using several methods such as Fourier transform-infrared (FT-IR), ultraviolet-visible (UV-VIS), X-ray photoelectron spectroscopy (XPS) and thermogravimetric analysis (TGA). Since MSN shows a lower drug release amount due to the agglomeration tendency, in order to increase MSN dispersion and drug release amount from MSN, two common biocompatible and biodegradable polymers were used as polymer matrix in which the MSN-BZC can be dispersed. The drug release profile of the MSN-BZC and of the synthesized hybrid materials were studied both in simulated gastric fluid (SGF) and simulated intestinal fluid (SIF). Polymer-MSN-BZC hybrid materials exhibit a higher drug release percent than the pure MSN-BZC when a higher dispersion is achieved. The dispersion of MSN into the hybrid materials was pointed out in scanning electron microscope (SEM) images. The release mechanism was determined using four mathematic models including first-order, Higuchi, Korsmeyer–Peppas and Weibull.

## 1. Introduction

In recent years, drug delivery systems have been rapidly developed and become an important field in medical applications [1,2]. Among them, oral administration is the most widely used system exhibiting many advantages including easily self-administration, painless and low cost [3].

Vallet-Regi proposed for the first time mesoporous silica nanoparticles (MSN) as controlled delivery systems [4]. Since then it was found that this type of material may be one of the greatest carrier materials for hydrophobic or hydrophilic drugs [5] due to their controlled pore size, large surface area, pore volume and, moreover, it has been demonstrated that it has very high biocompatibility both in vitro and in vivo [6]. Unlike other carriers, MSN exhibit a higher resistance to temperature and pH variation, mechanical stress and hydrolysis-induced degradations thus making a stable and rigid framework. Another important advantage of MSN for the medical field is their degradability in aqueous solution which can avoid further problems related to the removal of the material after use, being easily excreted from the body. Since for the medical field it is very important to control the pore size, geometry and shape of the carrier, in the case of MSN the size and the surface chemistry of the pore could be easily controlled and changed depending on the drug which should be encapsulated to obtain the proper loading and release of the drug. Moreover, MSN proved to exhibit a higher versatility compared with other systems like polymer nanoparticles and liposomes.

However, despite the potential benefit of this carrier for drug delivery applications, some important challenges were reported. The agglomeration tendency of the particles exhibits a strong influence on the drug-loading capacity by decreasing the amount of drug loaded due to the steric hindrance. The agglomeration tendency is specific for inorganic nanoparticles and it is a very important factor that must be taken into account [7]. Although MSN were accepted as having a low toxicity and a good biocompatibility at nano-scale, some biocompatibility studies showed that MSN particles with diameters ranging from 150 nm have significant toxicity at high concentrations in vitro, and cause severe systemic toxicity in vivo after intraperitoneal and intravenous injections [8]. Synthesis of hybrid materials based on biopolymers and MSN may solve some of the aforementioned problems since polymer/silica composites may encapsulate large amounts of guest molecules and subsequently release them at later stages in an optimal way [9]. Biopolymers such as chitosan (CS) and alginate (Al) exhibit many advantages for developing an ideal drug delivery system [10,11]. They have high biocompatibility, biodegradability, bioadhesivity, antibacterial activity, etc. So the introduction of MSN within de polymer matrices will exceed their biocompatility for the human body.

Benzalkonium chloride (BZC), a quaternary ammonium salt with the general formula [C_6_H_5_CH_2_N(CH_3_)_2_R^+^]Cl^−^ where R range from C_8_H_17_ to C_19_H_39_ is a bacteriostatic agent used as a preservative and disinfectant in the pharmaceutical industry. It is often utilized as an antiseptic and medical equipment disinfectant similar to other cationic surfactants [12,13]. It has different physical, chemical and microbiological properties.

In the present work, the optimal parameters for BZC adsorption into mesoporous silica nanoparticles and its drug release are discussed. For that, the influence of the contact time, pH of the solution and temperature were considered. The adsorption mechanism of the drug has been determinated by using the equilibrium data. The MSN and BZC were further incorporated into two common biopolymers and the drug release profile and the release mechanism have been also pointed out. The biopolymers were chosen in order to decrease MSN agglomeration and increase the amount of drug release. The dispersion of MSN into the two biopolymers was observed into scanning elecron microscope (SEM) images for the synthesized hybrid materials.

## 2. Materials and Methods

### 2.1. Materials

BZC, CS with medium molecular weight, Al, glutaraldehyde (GA), calcium chloride used as gelling agents and mesoporous silica (MCM-41) with a pore size of about 2.1–2.7 nm, 0.98 cm^3^/g pore volume and a specific surface area ~1000 m^2^/g were purchased from Sigma Aldrich.

Sodium hydroxide, potassium phosphate monobasic, hydrochloric acid, potassium chloride were received from Sigma Aldrich.

### 2.2. Immobilization of Benzalkonium Chloride (BZC) to Mesoporous Silica Nanoparticles (MSN)

BZC was used as a model drug in order to evaluate the possible drug delivery capacity of different hybrid materials (Al-MSN; CS-MSN).

0.015 g BZC were dissolved in 2.5 mL pH 5 solutions and then 0.1 g of MSN were added under magnetic stirring. The stirring was maintained for 2 h at RT. The obtained suspension was centrifuged and dried at 35 °C for 24 h in a vacuum oven.

### 2.3. Synthesis of Chitosan (CS)-BZC and CS-MSN-BZC Hybrid Materials

50 mg of CS powder was dissolved in 10 wt.% acetic acid solution for 24 h at RT to form a homogenous viscous solution. The CS-BZC (20 mL CS and 0.015 g BZC) and CS-MSN-BZC (20 mL CS solution, 0.015 g BZC and 0.1 g MSN) suspension were obtained by mixing the two or three compounds and mechanically stirring at room temperature (RT) for 2 h. Then 0.0225 mL aqueous solution of GA (25 wt.%) was added as crosslinking agent and the stirring was continued for another one hour. CS-BZC and CS-MSN-BZC hybrid materials were cast onto transparent Petri dish and left undisturbed for 72 h at RT for solvent evaporation and thus, allowing to form thin films.

### 2.4. Synthesis of Alginate (Al)-BZC and Al-MSN-BZC Hybrid Materials

A solution of Al was prepared by dissolving 50 mg of Al in 50 mL of water for 3 h. The Al-BZC and Al-MSN-BZC suspensions were obtained following the same procedure described above (see Section 2.3). After solvent evaporation, the films were peeled off from the mold and impregnated in 1% CaCl_2_ aqueous solution for 1 h. The samples were washed several times with water to remove CaCl_2_ excess and dried for 24 h at RT.

### 2.5. Characterization

Fourier transform-infrared (FT-IR) measurements were performed on a Bruker VERTEX 70 spectrometer. The FT-IR spectra were recorded in 400 ÷ 4000 cm^−1^ range with 4 cm^−1^ resolution. The samples were analyzed from KBr pellets.

The X-ray photoelectron spectroscopy (XPS) spectra were registered on a Thermo Scientific K-Alpha equipment, fully integrated, with an aluminum anode monochromatic source. Charging effects were compensated by a flood gun. Pass energy of 200 eV and 20 eV were used for surgery and high resolution spectra aquisition respectively.

Thermogravimetric analysis (TGA) was done on a Q500 TA Instruments equipment. 2 mg of sample was heated from RT to 700 °C using a heating rate of 10 °C/min under constant nitrogen flow rate.

UV adsorption measurements of BZC were performedat λ = 262 nm on a UV 3600 Shimadzu equipment provided with aquartz cell having a light path of 10 mm.

The morphological characterization of the CS/Al-MSN-BZC composite films was evaluated from the micrograph recorded using a Philips Xl 30 ESEM TMP scanning electron microscope (SEM).

### 2.6. Adsorption Experiments

For adsorption experiments, the influences of contact time, temperature, concentration of the drug and buffer pH were investigated. To establish the influence of contact time on the BZC adsorption on MSN, 0.015 g BZC and 0.1 g MSN were mixed in various buffer solutions with pH 5 for 10, 30, 60, 120, 240, 360 min. The influence of the temperature reaction was studied using the same quantities of drug and MSN. The reactions were kept for 1 h, at room temperature, 40 °C, 60 °C and 80 °C. The effect of pH was studied by maintaining the temperature reaction for 1 h at 80 °C in solutions with different pH values: 3, 4, 5, 6, 7, 8, 9, 10, 11. Another important parameter that was studied was the initial drug concentration: 3 g/L, 6 g/L, 11 g/L, 18 g/L, 24 g/L, 36 g/L, 47 g/L, 100 g/L, 200 g/L.

The unabsorbed drug concentration was determined from ultraviolet (UV) spectra at 262 nm, after the centrifugation of MSN-BZC suspension. The amounts of drug adsorbed at time t (*Q_t_*, mg/g) and at equilibrium (*Q_e_*, mg/g) were calculated using the following equations:

(1) $$Q_{t}=\frac{(C_{0}-C_{t})V}{W}$$

(2) $$Q_{e}=\frac{(C_{0}-C_{e})V}{W}$$

where *C*_0_, *C_t_*, *C_e_* (mg/L) are the initial, the time and the equilibrium concentrations of BZC solution; *V* (L) is the volume of BZC solution, *W* (g) is the mass of MSN employed

### 2.7. In Vitro Drug Release Studies

The drug release studies were accomplished into a fully automated dissolution bath USP Apparatus 1 (708-DS Agilent) connected to an autocontrolled multi-channel peristaltic pump (810 Agilent) and at a UV-VIS spectrophotometer (Cary 60) with 1 mm flow cell and UV-Dissolution software. In a dialysis membrane bag was introduced certain amount of BZC-MSN, CS/Al-BZC and CS/AL-MSN-BZC hybrid materials and 4 mL buffer solution of pH 7.4 (simulated intestinal fluid, SIF) and pH 1.2 (simulated gastric fluid, SGF) respectively prepared as described by A. Ghebaur and coworkers [14]. These dialysis membranes were caught by the Apparatus 1 rods and immersed in 200 mL buffer solution.

The dissolution bath temperature was kept constant at 37 °C and the spindle rotation speed was set 100 rpm. At various time intervals the dissolution media were automatically extracted and the BZC concentration was calculated from the UV adsorption at 262 nm.

### 2.8. In Vitro Kinetic Evaluation

In vitro kinetic evaluation of BZC from different types of materials was analyzed by 4 various kinetic models: first order, Higuchi, Kormeyer–Peppas, Weibull.

*First order model* describes the release of drug from pharmaceutical forms that encapsulate water-soluble drugs in porous matrices [15] according to this model

(3) $$\log C=\log C_{0}-\frac{Kt}{2.303}$$

where *C*_0_ is the initial concentration of drug, *K* is the first order rate constant and *t* is the time. The obtained data are plotted as log cumulative percent of drug remaining vs. time. A straight line with the slope −*K*/2.303 will be obtained.

*Higuchi model* descries the release of water soluble and low soluble drugs from semisolid and/or solid matrices [16,17].

The equations that describes the Higuchi model is:

(4) $$Q=K_{H}\times t^{1/2}$$

where Q is the amount of drug release at time *t*, *K_H_* is the Higuchi dissolution constant. The data obtained are plotted as cumulative percentage drug release versus square root of time.

*Korsmeyer–Peppas model* describes the release of a drug from a polymeric system [18,19,20].

The equation for this model is:

(5) $$\frac{M_{t}}{M_{\infty }}=kt^{n}$$

where *M_t_*/*M*_∞_ is fraction of drug released at time *t*, *k* (min^−n^) is the release rate constant and *n* is the release exponent. The data obtained are plotted as log cumulative percentage drug release versus log time. The *n* value is used to determine the release mechanism (see Table 1).

The *Weibull model* is use to compare the release profile of different types of drug delivery matrixes [21]. The equation that describes this model is:

(6) $$\frac{M_{t}}{M_{\infty }}=1-\exp\left(-at^{b}\right)$$

where *M_t_* is accumulated fraction of drug in solution at time *t*, *M*_∞_ is total amount of drug being released, *a* is the scale parameter that defines the time scale process, *b* is the parameter that describes the shape of the dissolution curve progression. For *b* = 1 the curve shape is exponential, *b* > 1 the curve shape is sigmoidal and if *b* < 1 the curve shape is parabolic.

## 3. Results and Discussion

### 3.1. Characterization of the Modified MSN with BZC

FT-IR analysis

**Figure 1:**
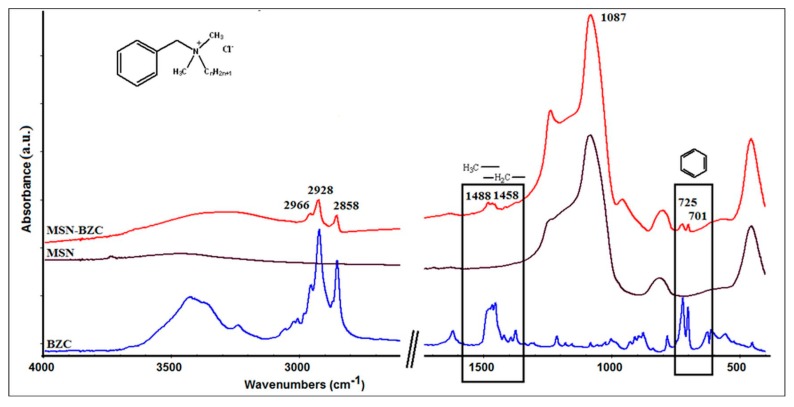
The Fourier transform-infrared (FT-IR) spectra for mesoporous silica nanoparticles (MSN), benzalkonium chloride (BZC) and MSN modified with BZC.

The FT-IR spectra were recorded to confirm the interaction between MSN and BZC (see Figure 1). The MSN spectrum exhibits the bands at 453 cm^−1^, 861 cm^−1^, 1087 cm^−1^ and 1643 cm^−1^ assigned to the characteristic vibrations of the silica substrate [22]. The band at 1643 cm^−1^ can be attributed to the adsorbed water molecules while the bands at 453 cm^−1^, 861 cm^−1^, 1087 cm^−1^ were assigned to Si-O-Si bending and stretching vibrations. From the FT-IR spectrum of modified MSN with BZC some additional peak can be observed which confirm the presence of BZC. Thus the bands from 2966 cm^−1^, 2928 cm^−1^ and 2858 cm^−1^ are attributed to symmetric and asymmetric stretching vibration of the C–H bond of the BZC tail. The peak from 701 cm^−1^ corresponds to the C-H bending vibration from aromatic ring while the 1458 cm^−1^ and 1488 cm^−1^ peaks are assigned to the C-H bending from methyl (-CH_3_) and methylene (-CH_2_) groups [12].

XPS analysis

**Figure 2:**
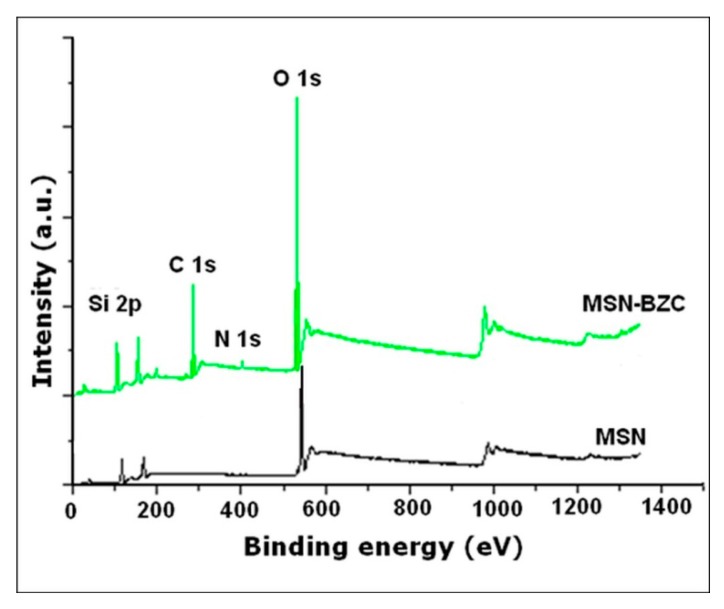
The X-ray photoelectron spectroscopy (XPS) survey spectra for MSN and modified MSN with BZC.

The XPS analysis of MSN modified with BZC was done to show the chemical composition of the surface after the modification process (Figure 2). All the samples show the Si 2p and O 1s peaks which are assigned to silica framework. In the XPS survey spectra of the modified MSN, the presence of C 1s (BE = 282 eV) and N 1s (BE = 402 eV) can be clearly observed indicating that the modification process occurred.

TGA data

**Figure 3:**
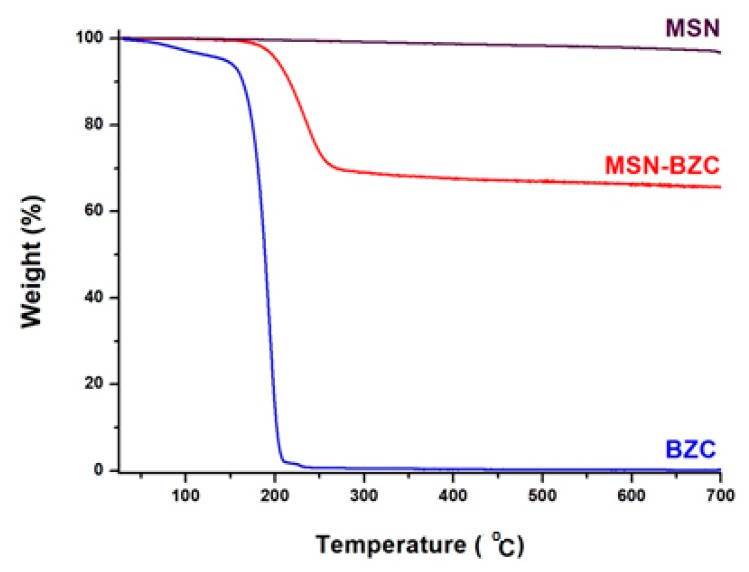
Thermogravimetric analysis (TGA) curves for MSN, BZC and MSN modified with BZC.

The adsorption of BZC onto MSN was also confirmed from TGA curves (Figure 3). The MSN modified with BZC shows two thermal decomposition steps. The first step, around 200 °C, is attributed to the thermal degradation of the loaded drug and the second step which occurs at a higher temperature, around 300 °C, is assigned to the thermal degradation of the inorganic fraction [23].

The MSN modified with BZC exhibits also an increase of weight loss compared to unmodified MSN which is due to the thermal degradation of the organic compound adsorbed onto the MSN surface or/and within MSN pores.

### 3.2. The Influence of Contact Time, pH, Temperature and Concentration of BZC

Contact time influence

**Figure 4:**
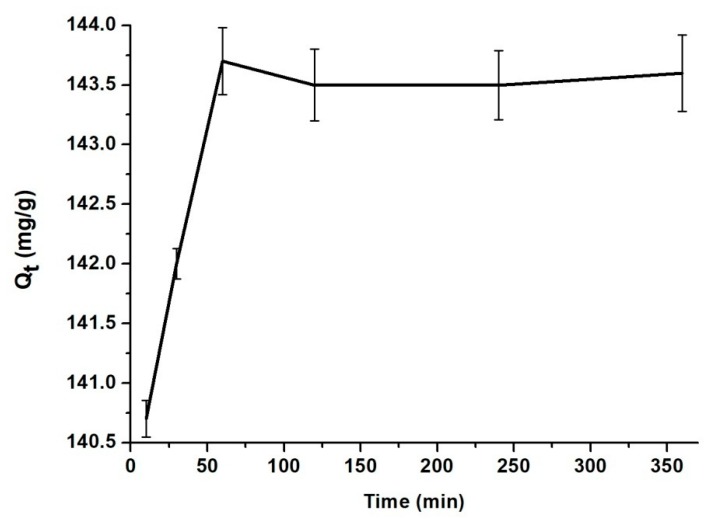
The influence of the contact time of BZC with MSN.

The effect of the contact time for the adsorption of BZC onto MSN is presented in Figure 4. MSN has a good adsorption capacity, within 60 min the equilibrium being achieved. There was no significant change between the samples from 1 h to 6 h.

In order to determine the adsorption process type and to predict the adsorption rate, the kinetic parameters were determined. The adsorption of BZC onto MSN has been calculated using the pseudo-second order equation:

(7) $$\frac{dq_{t}}{d_{t}}=k_{2}{\left(q_{e}-q_{t}\right)}^{2}$$

where *k*_2_ is the rate constant of second-order adsorption in (g mg^−1^ min^−1^).

Equation (7) can be integrated using boundary conditions *t* = 0 to *t* = t and *q* = 0 to *q* = q and gives:

(8) $$\frac{1}{\left(q_{e}-q\right)}=\frac{1}{q_{e}}+k_{2}t$$

Equation (8) can be linear, written as:

(9) $$\frac{t}{\left(q_{t}\right)}=\frac{1}{k_{2}\times q_{e}{}^{2}}+\frac{1}{q_{e}}t$$

The straight-line plots of (*t*/*q*) versus *t* have been drawn to obtain rate parameters, *k*_2_ and *q_e_* [24].

The high correlation coefficient (R^2^ = 1) suggests that the adsorption process of BZC onto MSN follows the pseudo-second-order kinetic model (see Table 2). Also, the calculated *q_e_* has almost the same value as the *q_e_* determined experimental [25].

The temperature influence

**Figure 5:**
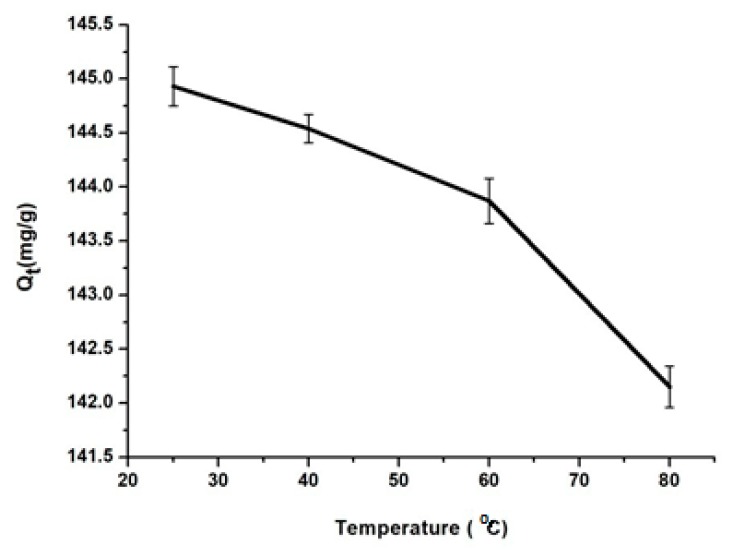
Adsorption of BZC at different temperature onto MSN.

The temperature effect on drug adsorption onto MSN was studied at RT, 40 °C, 60 °C and 80 °C. As can be observed in Figure 5, the highest amount of drug, 144.9 mg/g, was adsorbed at RT. At higher temperature values BZC starts too degraded, according to TG analysis (Figure 3).

Also, the thermodynamic parameters were calculated. The free energy (ΔG^0^), enthalpy (ΔH^0^) and entropy (ΔS^0^) were calculated using the following equations:

ΔG^0^ = −RT ln (Kc) (10)

ΔG^0^ = ΔH^0^ − TΔS^0^ (11)

Kc = Q*e*/C*e* (12)

where Kc (L/g) is the adsorbed capacity to retain the active substance, R (8.314 J/mol K) is the universal gas constant and T (K) is the temperature. The values of ΔH^0^ and ΔS^0^ are calculated from the slope and intercept of the plot of ln (Kc) versus 1/T (Table 3). The negative values of the free energy indicate a spontaneous and physical process. This is confirmed by ΔH^0^ values, which are smaller than 25 kJ/mol [26]. The positive values of ΔS^0^ indicate a higher disorder of MSN as the adsorbed drug onto their surface increase. Since ΔG^0^ is negative and ΔS^0^ positive, the adsorption process is spontaneous with high affinity for BZC [27].

The pH influence

The pH value of the solution is one of the most important parameters for the adsorption of pharmaceutics onto mesoporous silica nanoparticles surface or pores. Solutions with the pH values in the range between 3 to 11 were employed, these values being adjusted with HCl 0.1 N or NaOH 0.1 N solutions.

**Figure 6:**
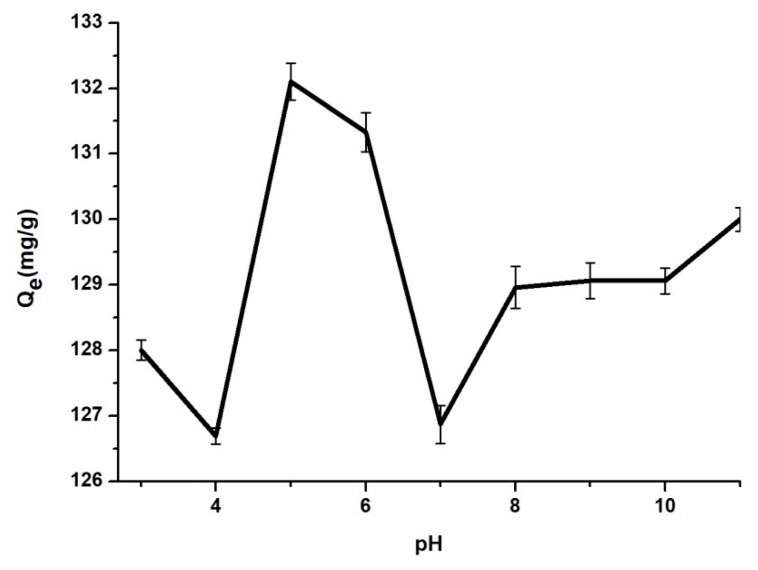
Adsorption of BZC at different pH values onto MSN.

The pH influence on the adsorption process of BZC onto the MSN surface or pores is shown in Figure 6. Thus, a BZC solution with an initial concentration of 6 g/L was used. Up to pH 5, the amount of the adsorbed BZC increases. After, a slowly decreases is observed to pH 6 and furthermore an abrupt decreases at higher pH values was noticed [28]. This phenomenon takes place because, at this concentration, the pH solution is 5–6 and the drug solubility is maximum [29]. Also, the molecule activity in solvent at this concentration and pH value is maximum.

The influence of initial drug concentration

**Figure 7:**
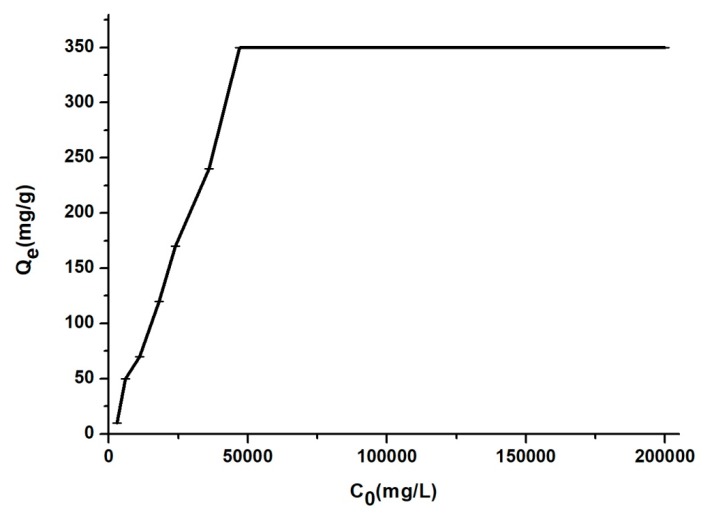
The influence of drug concentration onto MSN.

Figure 7 illustrates the effect of initial BZC concentration against adsorption onto MSN. An increase of the equilibrium values from 18.56 to 352 mg/g for the adsorbed amount of the drug onto MSN was noticed when the initial drug concentration was increased from 3000 to 50,000 mg/L. For values of C_0_ higher than 50,000 mg/L, the values of q_e_ are almost unchanged.

### 3.3. In Vitro Release Studies

**Figure 8:**
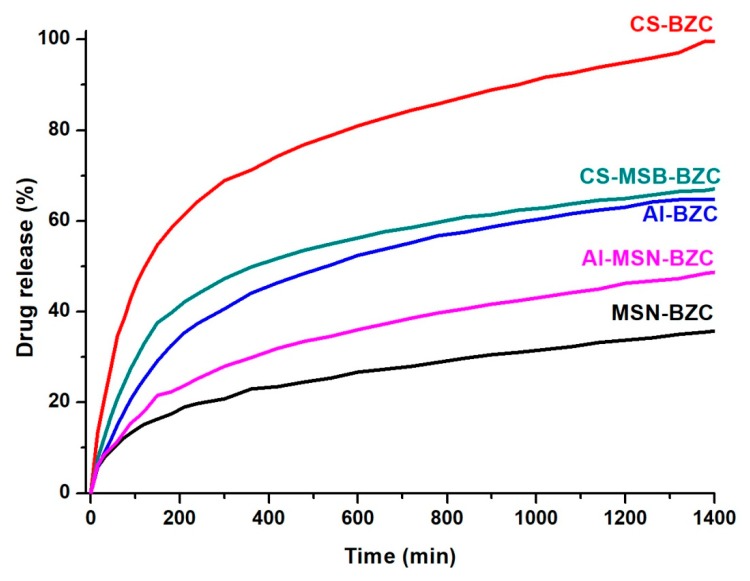
The release profile of BZC from different hybrid materials in simulated gastric fluid (SGF).

**Figure 9:**
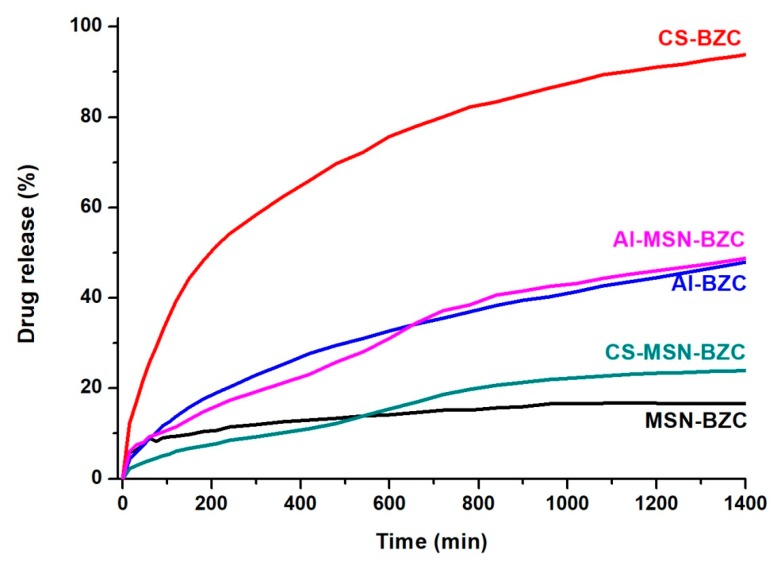
The release profile of BZC from different hybrid materials in simulated intestinal fluid (SIF).

In vitro release of BZC was investigated both in SIF (pH 7.4) and SGF (pH 1.2). Figure 8 and Figure 9 show the amount of BZC released from MSN, from the two biopolymers and from the corresponding hybrid systems. At pH 1.2 (Figure 8), the systems CS-BZC and CS-MSN-BZC release a higher amount of drug than Al-BZC and Al-MSN-BZC systems due to the high swelling degree of CS in SIF [30]. At pH 7.4, the amount of the drug released within 24 h from the CS was higher than for Al also due to the good swelling behavior of the biopolymer in SIF which favors the diffusion of the drug molecules. Conversely, the lower release amount of BZC from Al matrix was attributed to a higher crosslinking density of the polymer which slows down considerably the diffusion of the drug and inhibits the crossing of water molecules through the polymer chains. These results are in good agreement with Xiujuan Huang and coworkers who report that the delivery of the drug might be determinate by varying the amount of sodium alginate and CaCl_2_ concentration [30]. Moreover, Figure 8 and Figure 9 show that the amount of BZC released from MSN-BZC systems is lower than the amount of drug released from the CS-MSN-BZC and Al-MSN-BZC hybrid materials. These results were attributed to a good dispersion of MSN in polymer matrices which reduced nanoparticles agglomeration and enabled a better drug diffusion.

### 3.4. Scanning Electron Microscopy (SEM) Analysis

**Figure 10:**
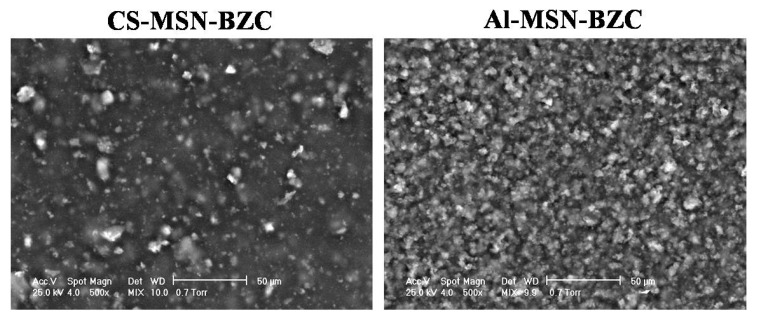
Scanning electron microscope (SEM) images recorded on the CS-MSN-BZC and AL-MSN BZC films.

In order to prevent the already reported agglomeration and sedimentation of MSN into aqueous solution [31], MSN was dispersed into the polymer matrix to achieve a more stable colloidal system. The dispersion of MSN into the polymer matrix was investigated by SEM. Figure 10 displays the surface morphology of both CS-MSN-BZC and Al-MSN-BZC films. According to the micrographs, the MSN dispersion in CS and Al is different. While a good dispersion is observed for Al-MSN-BZC, in the case of CS-MSN-BZC agglomerates are noticed. This result supports our initial claim, that a better MSN-BZC dispersion will facilitate the drug release and explains the higher drug release percent registered for Al-MSN-BZC in comparison with the CS-MSN-BZC hybrid material.

### 3.5. In Vitro Kinetic Evaluation

The release mechanism of drug from different type of materials depends by the physic-chemical properties of host materials and the pore size from the materials or by the size of microparticles or nanoparticles [32]. Four types of mathematic models were used in this paper to determine the release mechanism of BZC from MSN-BZC, AL-BZC, CS-BZC, AL-MSN-BZC and CS-MSN-BZC in two different release media (pH 1.2 and pH 7.4). The calculated parameters from these models are presented in Table 4. The mathematic model which shows a correlation coefficient (R) near 1 is the model that is suitable to characterize the release mechanism.

The release of BZC from polymer obeys the Weibull model in pH 1.2 with a R2 values of 0.9974 for CS-BZC and 0.9875 for Al-BZC meaning that the properties of the release medium have a high impact on drug release. Al tends to be more stable at pH 1.2 compared with CS. At pH 7.4, the release of BZC obeys the first order or Higuchi model having a coefficient R2 value of 0.991 for CS-BZC and of 0.9681 for Al-BZC, meaning that the drug was transported from the matrix by diffusion for both CS and Al.

The introduction of MSN in the polymer matrix does not induce a significant modification in the release mechanism. At pH 1.2, the Weibull or Higuchi model describes the drug release and also the diffusion is the transport mechanism due to the different stability of the two polymers in SGF. Conversely, at pH 7.4 the mathematic model that describes the release mechanism is the first order model meaning that MSN induce its porosity in the hybrid material. The results obtained fit well the synthesized materials because the model is similar to a drug delivery system that contains watersoluble drugs which are encapsulated into porous matrices [33]. This time the drug respects the case II mechanism transport from the hybrid material (swelling followed by erosion).

## 4. Conclusions

The successful modification of MSN with different amounts of BZC was proved by FT-IR spectra in which distinctive bands assigned to BZC structure into the MSN-BZC spectra were identified, as well from the TGA data where a significant mass loss is obtained for MSN-BZC compared with pure MSN. Moreover, XPS was used to confirm the presence of BZC on the MSN surface.

The pH value of the environment, the contact time or the temperature used during the adsorption experiments proved to be important factors in the encapsulation process of BZC into the MSN. The highest encapsulation degree was recorded in solution having pH value of 5, at room temperature when the MSN was immersed for 60 min into the BZC containing solution. The adsorption process type as well as the adsorption rate, the kinetic parameters and thermodynamic parameters were determined.

Using a casting method, we obtained CS/AL-BZC and CS/AL-MSN-BZC composite films, which were further tested as potential drug delivery systems. The release profile of BZC from different systems, was studied in both SGF and SIF. The biopolymers are intended to increase the amount of the drug release by improving the dispersion of the MSN and allow a better diffusion of the drug. This is evident from the drug release curves of the MSN-BZC hybrid materials with and without the polymer. SEM proved the dispersion of the MSN within the polymer matrices whereby we observed a good distribution of the inorganic filler within the Al-MSN-BZC and the formation of some agglomeration in the case of CS-MSN-BZC. The dissolution media and the presence of MSN in the polymer matrix significantly influence the release mechanism. In SIF the release mechanism of BZC obeys the first order model because MSN increases the porosity of the hybrid materials.

## Figures and Tables

**Table 1:** Interpretation of drug transport mechanism for film-like materials.

n	Transport mechanism
0.5	Fickian diffusion
0.5 < n < 1	Anomalous Transport
1	Case II transport
1 < n	Super case II transport

**Table 2:** Kinetic parameters for BZC adsorption onto MSN.

Kinetic Model	
**Pseudo-Second Order**	
*q_e_*, calc (mg/g)	142.85
*k*_2_ (g/mg min)	0.037692
R2	1
**Intr-Particle Diffusion**	
k_p_ (mg/g min)	0.6245
R2	0.9914

**Table 3:** Thermodynamic parameters for BZC adsorption onto MSN.

Temperature (K)	298	313	333	353
ΔG^0^ (kJ/mol)	−2.3	−2.77	−3.4	−4.02
ΔH^0^ (kJ/mol)	7.04			
ΔS^0^ (kJ/molK)	0.031			

**Table 4:** Kinetic release parameters of BZC release process from MSN-BZC, AL-BZC, CS-BZC, AL-MSN-BZC, CS-MSN-BZC in different simulated body fluids.

Sample		First Order			Higuchi		Korsmayer-Peppas			Weibull		
		C_0_, mg/g	K, h^−1^	R^2^	K_H_, h^−1/2^	R^2^	K (min^−n^)	n	R^2^	a	b	R^2^
MSN-BZC	pH 1.2	94.8	0.00069	0.89652	0.138	0.9339	0.4428	0.3848	0.9923	0.001	0.0024	0.9754
	pH 7.4	93.22	1.15 × 10^−6^	0.9007	0.093	0.8327	0.4203	0.2916	0.9337	0.0005	0.0011	0.8017
AL-BZC	pH 1.2	98.81	0.00184	0.9796	0.2925	0.9456	0.4913	0.5	0.9594	0.0023	0.0067	0.9875
	pH 7.4	90.78	0.00046	0.9681	0.1951	0.9976	0.9806	0.5379	0.9964	0.0017	0.0054	0.9466
CS-BZC	pH 1.2	83.69	0.00276	0.885	0.526	0.8266	0.1418	0.3782	0.9419	0.0034	0.0079	0.9974
	pH 7.4	96.74	0.00184	0.991	0.433	0.9442	0.2283	0.4401	0.9801	0.0031	0.0085	0.9845
AL-MSN-BZC	pH 1.2	96.83	0.001152	0.9407	0.2026	0.9795	0.5025	0.45	0.9892	0.0016	0.0046	0.9736
	pH 7.4	96.6	0.00046	0.88	0.1684	0.8716	1.7196	0.5924	0.9559	0.0015	0.0055	0.7707
CS-MSN-BZC	pH 1.2	74.1	0.00069	0.8743	0.6255	0.8451	0.2614	0.4146	0.9304	0.0043	0.0106	0.9949
	pH 7.4	97.19	1.15 × 10^−9^	0.977	0.9418	0.9555	3.8884	0.6314	0.9911	0.0098	0.0376	0.8927
